# Trends in Sentinel Lymph Node Biopsy Enactment for Cutaneous Melanoma

**DOI:** 10.1245/s10434-019-07204-2

**Published:** 2019-02-04

**Authors:** Mary-Ann El Sharouni, Arjen J. Witkamp, Vigfús Sigurdsson, Paul J. van Diest

**Affiliations:** 1 Department of Dermatology, University Medical Centre Utrecht, Utrecht University, Utrecht, The Netherlands; 2 Department of Surgery, University Medical Centre Utrecht, Utrecht University, Utrecht, The Netherlands; 3 Department of Pathology, University Medical Centre Utrecht, Utrecht University, Utrecht, The Netherlands

## Abstract

**Background:**

Over recent years, sentinel lymph node biopsy (SLNB) recommendations in guidelines for cutaneous melanoma have changed considerably. We aimed to assess trends in enactment of SLNB to evaluate to what extent guidelines were adhered to, and to identify clinical and pathological determinants of (non-)adherence.

**Methods:**

Clinicopathological data from the Dutch nationwide network and registry of histopathology and cytopathology were retrieved from patients diagnosed with primary cutaneous melanoma in The Netherlands between 2003 and 2014. SLNB enactment was analyzed per year. Multivariable regression models were developed to assess the determinants of SLNB enactment.

**Results:**

A total of 51,510 primary cutaneous melanomas in 49,514 patients were diagnosed, of which 24,603 melanomas were eligible for SLNB as they were staged T1b or higher. In practice, only 9761 (39.7%) patients underwent SLNB, with an increasing trend from 39.1% in 2003 to 47.8% in 2014 (*p* < 0.001). A total of 759 (2.9%) of 26,426 patients without SLNB indication underwent SLNB anyway. Variables significantly associated with enactment of SLNB were male sex, younger age, and melanoma on sites other than the head and neck.

**Conclusions:**

Although there was an increasing trend in time in SLNB enactment, enactment of SLNB did not comply well with recommendations in (inter)national guidelines. Female sex, higher age, and melanoma located on the head and neck were associated with non-enactment of SLNB.

Melanoma accounts for the vast majority of skin cancer-related deaths (almost 90%)^1^,^2^ mainly due to regional or distant metastases that have already formed by the time of diagnosis. Reasoning from the concept of a stepwise spread of metastases through locoregional lymph nodes before going into the bloodstream to distant sites, locoregional lymph node dissection was introduced as a therapeutic procedure. However, for many sites of the body (apart from the extremities), the nearest lymph node basin is not obvious, making it often difficult to decide where to perform lymph node dissection and failing to improve prognosis. With the introduction of the sentinel lymph node biopsy (SNLB) procedure to identify the exact location of the first draining lymph node,^3^ it became possible to both perform a targeted lymph node dissection aiming to improve the prognosis of SLNB-positive patients, and deny SLNB-negative patients a superfluous surgical procedure. Following its introduction, various guidelines around the world incorporated SNLB indications to select which patients would benefit and which patients would not. For the first time, in 1997, the Dutch national melanoma guidelines mentioned SLNB as ‘a promising intervention’.^4^ On an international level, the 6th edition of the American Joint Committee on Cancer (AJCC) staging manual (2003–2009) incorporated the SLNB result into the definition of pathological staging.^5^ Indications for SLNB in the 6th AJCC were not specifically defined per stage as SLNB was “a standard for staging nodal metastases in patients with clinically uninvolved lymph nodes”.^6^ Although (inter)national guidelines differed slightly, most agreed that in the 7th AJCC, an indication for SLNB was melanoma with a Breslow thickness (BT) > 1.00 mm, with some guidelines also including select patients with a BT ≤ 1.00 mm (e.g. when other adverse parameters such as ulceration, increased mitotic rate, or young age were present).^7^–^9^ Various follow-up studies have shown that SLNB status can provide important prognostic information,^10^–^14^ but, in 2006, the results from the Multicenter Selective Lymphadenectomy Trial (MSLT)-1 showed that SLNB has no therapeutic value as it did not seem to improve melanoma-specific survival.^13^ Anticipating this publication, guidelines were adapted to no longer propose routine SLNB, but they advised to enact SLNB to inform selected groups of patients on their prognosis. Furthermore, the MSLT-II trial showed that additional complete lymph node dissection does not increase melanoma-specific survival among patients with sentinel node metastases.^14^ The 2005 Dutch national guideline stated SLNB needs to be reserved for patients who want to be informed as optimally as possible, not as a standard diagnostic procedure.^15^ In 2007, guidelines were adapted to include the MSLT-1 results, without rectification of the aforementioned advice. Since 2012, Dutch guidelines have recommended SLNB as a prognostic procedure for patients with melanoma stage T1b or higher;^16^,^17^ however, the definition of stage T1 has changed over the years. Although BT cut-offs are equal at 1.00 mm, in the 6th AJCC ulceration was incorporated for the first time, and, in the 7th AJCC, mitoses ≥ 1/mm^2^ were added as a second determinant (besides ulceration) for T1a and T1b melanoma. In the recent 8th edition of the AJCC staging manual, mitoses were again eliminated.^18^

In view of these evolving views on the indications of SLNB as a staging or therapeutic procedure, the changes in the AJCC staging system, and less belief in a stepwise pattern of metastases, enactment of SLNB may well have changed over the years. The aim of this study was therefore to evaluate trends in enactment of SLNB in The Netherlands and analyze clinicopathologic determinants of (non-)adherence to guidelines.

## Methods

### Collection of Data

Data for this retrospective nationwide study were derived from ‘PALGA’, the Dutch nationwide network and registry of histopathology and cytopathology, which has prospectively collected all pathology data from all pathology laboratories in The Netherlands since 1987 (http://www.palga.nl).

### Study Population

For this cohort study, the pathologic reports of all newly diagnosed adult melanoma patients in The Netherlands between 2003 and 2014 were analyzed; for these patients, the 6th AJCC was valid from 2003 to 2009, and the 7th AJCC was valid from 2010 to 2014. Melanoma in situ, Spitzoid tumors of unknown malignant potential (STUMP), melanocytic tumors of unknown malignant potential (MELTUMP), and superficial atypical melanocytic proliferation of uncertain significance (SAMPUS) were excluded, as well as non-cutaneous, desmoplastic melanomas, and melanomas without, or unclear, BT reported. We excluded patients with a positive direct complete lymph node dissection, fine-needle aspiration, or otherwise diagnosed positive lymph nodes within 14 days of diagnosis of the melanoma to ensure patients were free of metastases prior to their melanoma. For the present study, this yielded a dataset of adults with histologically proven invasive, primary, cutaneous melanomas diagnosed between 2003 and 2014 in The Netherlands.

For each patient, clinicopathological variables were extracted from the pathology text files, including date of diagnosis, age, sex, BT (mm), T stage, ulceration (present or absent), type of melanoma (superficial spreading [SSM], nodular [NM], lentigo maligna [LMM], or acral lentiginous [ALM]), body site (head and neck, trunk, arms, or legs) and SLNB enactment (yes or no). As guidelines do not comment on the time between primary excision and SLNB, in a multidisciplinary setting, we decided to include all SLNBs performed within 100 days after initial diagnosis as SLNB, as previously described.^19^ Mitoses were included for melanoma for the time period the 7th AJCC was valid, since mitotic rate ≥ 1/mm^2^ implies SLNB indication. If patients had more than one primary melanoma, these melanomas were considered separately in the analysis, resulting in total number of melanomas instead of patients. SLNB guideline indication adherence was analyzed per year.

### Statistical Analysis

Univariate variables were analyzed using the Chi square test or Mann–Whitney U test, as appropriate. Continuous variables are presented as median with interquartile range (IQR) or mean with standard deviation (SD) for non-normally distributed data and normally distributed data, respectively. Categorical variables are presented as numbers and percentages. To prevent confounding by indication, patients with other lymph node-related procedures, such as complete lymph node dissection or fine-needle aspiration, within 100 days after initial melanoma diagnosis were excluded when calculating SLNB percentages and trends. Trends in time were assessed using a linear-by-linear association test. To account for a possible delay in adoption of the 7th AJCC guideline, we applied the 6th edition of the AJCC staging manual to the time period in our study for which the new 7th AJCC was applicable (2010–2014), leading to an additional analysis that excluded mitotic rate as a criterion. Regression models for melanoma with and without SLNB indication were developed to assess the association of clinicopathological variables (age [continuous], sex, BT [continuous for the model with SLNB indication, categorical for the model without SLNB indication], year [continuous], ulceration, body site, and melanoma subtype) with SLNB use. Variables were entered in a backward, stepwise method, and data were analyzed using SPSS version 21 (IBM Corporation, Armonk, NY, USA). Two-sided *p*-values < 0.05 were considered significant.

### Ethical Approval

All data were encoded and used anonymously. Ethical approval was granted by the board of PALGA.

## Results

### Patients and Melanoma Incidence Trends

Between 2003 and 2014, a total of 51,510 melanomas in 49,514 patients were diagnosed—47,549 single melanomas and 3961 multiple melanomas. According to AJCC staging, a total of 25,137 (48.8%) melanomas were staged to the 6th AJCC, and 26,373 (51.2%) were staged to the 7th AJCC. The total number of melanomas diagnosed per year increased from 2960 in 2003 to 5807 in 2014, with a median BT of 0.89 (IQR 0.50–1.70). A total of 55.4% of patients were female. Age ranged from 18 to 106 years, with a mean age of 56.98 years (SD 16.00). The trunk was the most common body site, harboring 42.6% of melanomas. Ulceration occurred in 6760 (13.1%) melanomas, and most melanomas were staged T1 (Table 1).

**Table 1:** Clinical and histopathological characteristics of all primary, cutaneous melanomas in The Netherlands from 2003 to 2014, with and without SLNB indication, stratified for enactment of SLNB

	All patients [*n* = 51,510]	Melanoma with SLNB indication [*n* = 24,603]^a^			Melanoma without SLNB indication [*n* = 26,426]		
		No SLNB enacted [*n* = 14,842]	SLNB enacted [*n* = 9761]	*p* value	No SLNB enacted [*n* = 25,667]	Enacted SLNB [*n* = 759]	*p* value
Sex [*n* (%)]				< 0.001*			0.67
Female	28,524 (55.4)	7919 (53.4)	4913 (50.3)		15,081 (58.8)	440 (58.0)	
Male	22,986 (44.6)	6923 (46.6)	4848 (49.7)		10,586 (41.2)	319 (42.0)	
Age, years							
Mean (SD)	56.98 (16.00)	62.28 (16.92)	54.98 (14.41)	< 0.001*	54.81 (15.36)	50.34 (13.48)	< 0.001*
*N* (%)				< 0.001*			< 0.001*
18–35	5042 (9.8)	1011 (6.8)	1001 (10.3)		2890 (11.3)	106 (14.0)	
36–55	18,658 (36.2)	4050 (27.3)	3832 (39.3)		10,258 (40.0)	379 (49.9)	
56–75	20,770 (40.3)	6006 (40.5)	4247 (43.5)		10,042 (39.1)	254 (33.5)	
> 75	7040 (13.7)	3775 (25.4)	681 (7.0)		2477 (9.7)	20 (2.6)	
Year of diagnosis [*n* (%)]				< 0.001*			< 0.001*
2003	2960 (5.7)	852 (5.7)	548 (5.6)		1463 (5.7)	66 (8.7)	
2004	3115 (6.0)	907 (6.1)	470 (4.8)		1646 (6.4)	57 (7.5)	
2005	3442 (6.7)	1019 (6.9)	494 (5.1)		1832 (7.1)	58 (7.6)	
2006	3462 (6.7)	1032 (7.0)	524 (5.4)		1816 (7.1)	56 (7.4)	
2007	3762 (7.3)	1056 (7.1)	635 (6.5)		1981 (7.7)	56 (7.4)	
2008	4085 (7.9)	1070 (7.2)	660 (6.8)		2247 (8.8)	67 (8.8)	
2009	4312 (8.4)	1086 (7.3)	768 (7.9)		2311 (9.0)	98 (12.9)	
2010	4693 (9.1)	1410 (9.5)	866 (8.9)		2315 (9.0)	63 (8.3)	
2011	5055 (9.8)	1505 (10.1)	985 (10.1)		2447 (9.5)	69 (9.1)	
2012	5260 (10.2)	1627 (11.0)	1066 (10.9)		2472 (9.6)	51 (6.7)	
2013	5557 (10.8)	1675 (11.3)	1280 (13.1)		2499 (9.7)	58 (7.6)	
2014	5807 (11.3)	1603 (10.8)	1465 (15.0)		2638 (10.3)	60 (7.9)	
Breslow thickness, mm							
Median (IQR)	0.89 (0.50–1.70)	1.60 (1.10–3.00)	1.85 (1.30–2.90)	< 0.001*	0.55 (0.40–0.70)	0.90 (0.73–1.00)	< 0.001*
*N* (%)				< 0.001*			NA
0.01–1.00	29,957 (58.2)	3012 (20.3)	513 (5.3)		25,667 (100.0) NA: T1a or T1NOS	759 (100.0) NA: T1a or T1NOS	
1.01–2.00	11,345 (22.0)	6203 (41.8)	5043 (51.7)				
2.01–3.00	4470 (8.7)	2244 (15.1)	2137 (21.9)				
3.01–4.00	2247 (4.4)	1188 (8.0)	985 (10.1)				
> 4.00	3491 (6.8)	2195 (14.8)	1083 (11.1)				
Body site [*n* (%)]				< 0.001*			< 0.001*
Head and neck	6412 (12.4)	2794 (18.8)	582 (6.0)		2891 (11.2)	42 (5.6)	
Trunk	21,937 (42.6)	5428 (36.6)	4286 (44.0)		11,703 (45.6)	324 (42.7)	
Arms	7637 (14.8)	2228 (15.0)	1469 (15.1)		3772 (14.7)	121 (15.9)	
Legs	13,953 (27.1)	3925 (26.4)	3159 (32.2)		6495 (25.3)	251 (33.1)	
Missing	1571 (3.0)	467 (3.2)	265 (2.7)		806 (3.1)	21 (2.8)	
Ulceration [*n* (%)]				< 0.001*			NA
No	38,463 (74.7)	9405 (63.4)	6539 (67.0)		NA: T1a or T1NOS	NA: T1a or T1NOS	
Yes	6760 (13.1)	3968 (26.7)	2545 (26.1)				
Missing	6287 (12.2)	1469 (9.9)	677 (6.9)				
Mitosis [*n* (%); 7th AJCC]	*N *= 26,373	*N* = 7820	*N* = 5663	< 0.001*			NA
No	8305 (31.5)	4288 (54.8)	2736 (48.3)		NA: T1a or T1nos	NA: T1a or T1nos	
Yes	7100 (26.9)	382 (4.9)	336 (5.9)				
Missing	10,968 (41.6)	3150 (40.3)	2591 (45.8)				
Subtype [*n* (%)]				< 0.001*			< 0.001*
SSM	37,163 (72.1)	8807 (59.4)	5991 (61.4)		21,562 (84.0)	618 (81.4)	
NM	6590 (12.8)	3510 (23.7)	2509 (25.7)		330 (1.3)	20 (2.6)	
LMM	2406 (4.7)	620 (4.2)	86 (0.9)		1682 (6.6)	13 (1.7)	
ALM	406 (0.8)	173 (1.2)	162 (1.6)		61 (0.3)	4 (0.5)	
Missing	4945 (9.6)	1732 (11.7)	1013 (10.4)		2032 (7.9)	104 (13.7)	
T-stage [*n* (%)]				< 0.001*			NA
T1	29,956 (58.2)	3012 (20.3)	512 (5.2)		NA: T1a or T1NOS	NA: T1a or T1NOS	
T2	11,334 (22.0)	6206 (41.8)	5043 (51.7)				
T3	6718 (13.0)	3433 (23.1)	3122 (32.0)				
T4	3492 (6.7)	2195 (14.8)	1084 (11.1)				

^a^ Exclusion of 481 patients with lymph node procedures other than SLNB

*SLNB* sentinel lymph node biopsy, *SD* standard deviation, *IQR* interquartile range, *AJCC* American Joint Committee on Cancer, *SSM* superficial spreading melanoma, *LMM* lentigo maligna melanoma, *ALM* acral lentiginous melanoma, *NM* nodular melanoma, *NA* not applicable, * indicates statistical significance

### Trends in Sentinel Lymph Node Biopsy (SLNB) Enactment

**Fig. 1:**
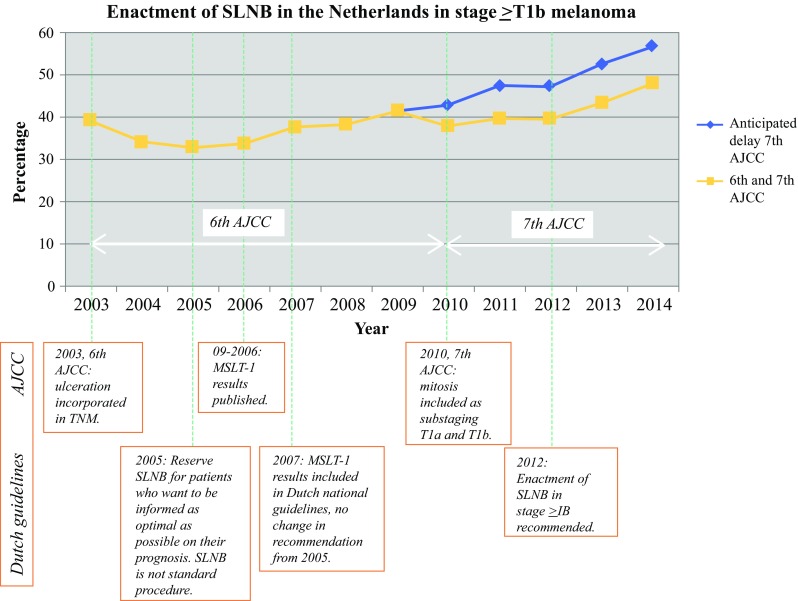
Trends in enacted SLNB (*n* = 9761) in ≥ T1b melanoma between 2003 and 2014 in The Netherlands, including the anticipated delay period of the 7th AJCC. Linear-by-linear association: *p < 0.001. *SLNB* sentinel lymph node biopsy, *AJCC* American Joint Committee on Cancer, *MSLT*-*1* Multicenter Selective Lymphadenectomy Trial-1

**Fig. 2:**
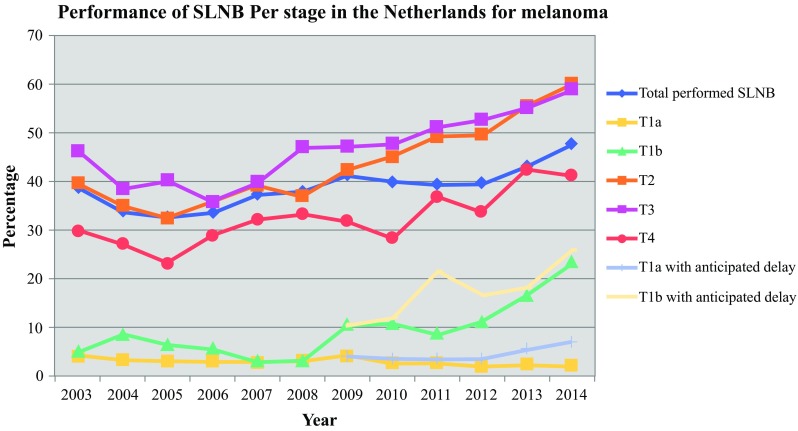
Percentage of enacted SLNB (*n* = 10,520) per stage per year for primary, cutaneous melanoma in The Netherlands, including the anticipated delay period of the 7th AJCC. Linear-by-linear association: *p < 0.001. *SLNB* sentinel lymph node biopsy, *AJCC* American Joint Committee on Cancer

The trend in time of SLNB enactment increased significantly from 39.1% in 2003 to 47.8% in 2014 (Fig. 1). When stratifying for T stage, we observed a trend for all stages, except T1a melanoma, especially from 2006 onward (Fig. 2). Adjusting the 100-day threshold for SLNB enactment to 200 days did not significantly alter these percentages (data not shown). When accounting for a possible delay to adoption of the 7th AJCC, 56.6% (instead of 47.8%) of SLNB enactments in all eligible patients (≥ T1b) would be reached in 2014, due to 2934 melanomas with mitoses > 1/mm^2^ that would have been staged T1b in the 7th AJCC, and in whom SLNB was not performed, but were classified as T1a in the 6th AJCC (Figs. 1 and 2).

### SLNB Enactment in Eligible Melanomas According to Guidelines

A total of 25,084 melanomas had an indication for SLNB, according to the guidelines at the time, as they were staged T1b or higher. Lymph node procedures other than SLNB were performed in 481 melanomas (1.9%). Excluding this group, a total of 9761 (39.7%) of all 24,603 eligible melanomas underwent SLNB in practice.

According to the evolving guidelines over the year, 14,842 melanomas had an indication for SLNB but were not enacted. This group had different characteristics than the group of melanomas in whom SLNB was enacted according to guidelines. Univariable analysis revealed that melanomas with SLNB indication but with no SLNB enactment had a higher mean age, comprised more females, had lower BT, and were less often ulcerated (Table 1). Multivariable analysis for enactment of SLNB excluded 4886 cases with missing values. All variables, except ulceration, showed a significant association with SLNB enactment. Women had significantly lower odds of receiving SLNB compared with men (odds ratio [OR] 0.78, 95% confidence interval 0.73–0.83), as had older patients and head and neck melanomas. ALMs and NMs were more likely to receive SLNB compared with SSMs (Table 2).

**Table 2:** Multivariable regression for factors associated with enactment of SLNB for cutaneous, primary melanoma (*n* = 19,717) in patients with SLNB indication in The Netherlands between 2003 and 2014

	OR (95% CI)	*p* value
Age, per year	0.97 (0.97–0.98)	< 0.001*
Breslow thickness, per mm	1.06 (1.04–1.07)	< 0.001*
Year, per year	1.07 (1.06–1.08)	< 0.001*
Body site		
Head and neck	Reference	
Trunk	2.97 (2.65–3.33)	< 0.001*
Arms	3.05 (2.68–3.47)	< 0.001*
Legs	3.47 (3.08–3.91)	< 0.001*
Subtype		
SSM	Reference	
NM	1.27 (1.18–1.37)	< 0.001*
LMM	0.64 (0.50v0.82)	< 0.001*
ALM	1.39 (1.09–1.76)	0.007*
Sex		
Male	Reference	
Female	0.78 (0.73–0.83)	< 0.001*

Ulceration not significant

*SLNB* sentinel lymph node biopsy, *OR* odds ratio, *CI* confidence interval, *SSM* superficial spreading melanoma, *NM* nodular melanoma, *LMM* lentigo maligna melanoma, *ALM* acral lentiginous melanoma, * indicate statistical significance

### SLNB Enactment in Non-Eligible Melanomas According to Guidelines

Conversely, a total of 759 (2.9%) of 26,426 patients without SLNB indication underwent SLNB. Of these, 500 (65.9%) had stage T1a and 259 (34.1%) had stage T1NOS. Compared with other non-eligible patients in whom SLNB was not performed, patients with SLNB enactment had a lower mean age of 50.34 versus 54.81 years, a higher median BT of 0.90 versus 0.55, and melanomas that were less often located on the head and neck (all *p* < 0.001). In multivariable regression, 2868 melanomas were excluded because of missing data. Patients who underwent SLNB without indication were more frequently males of younger age, with higher BT and melanoma on sites other than the head and neck (Table 3).

**Table 3:** Multivariable regression for factors associated with enactment of SLNB for cutaneous, primary melanoma (*n* = 23,558) in patients without SLNB indication in The Netherlands between 2003 and 2014

	OR (95% CI)	*p* value
Age, per year	0.98 (0.97–0.99)	< 0.001*
Breslow thickness, mm		
0.01–0.24	Reference	
0.25–0.49	1.68 (0.60–4.69)	0.32
0.50–0.74	3.61 (1.33–9.80)	0.01*
0.75–1.00	24.86 (9.27–66.69)	< 0.001*
Body site		
Head and neck	Reference	
Trunk	1.70 (1.17–2.47)	0.005*
Arms	2.19 (1.46–3.29)	< 0.001*
Legs	2.20 (1.50–3.23)	< 0.001*
Sex		
Male	Reference	
Female	0.80 (0.67–0.95)	0.01*

Year and type melanoma not significant. Ulceration not applicable (T1a or T1NOS melanoma)

*SLNB* sentinel lymph node biopsy, *OR* odds ratio, *CI* confidence interval, * indicates statistical significance

## Discussion

Over recent years, SLNB recommendations in guidelines for cutaneous melanoma have changed considerably. Current and previous Dutch and international guidelines advise SLNB in melanoma stage IB or higher.^14^,^20^–^22^ Dutch guidelines from 2005 describe SLNB as promising and to reserve it for patients who want to be ‘informed as optimally as possible’.^15^ In 2007, the results of MSLT-1 were included, without rectification of advice from 2005.^9^ From 2012, SLNB has been advised in all patients with melanoma stage T1b or higher.^16^,^17^

We have shown that in The Netherlands, the use of SLNB for melanoma has increased, likely due to these evolving guidelines following the results of landmark studies. Enactment of SLNB increased from 39.1% in 2003 to 47.8% in 2014. SLNB guidelines were apparently not adequately adhered to in The Netherlands as only 39.7% of eligible tumors underwent SLNB. Although an obvious increasing trend has been observed since publication of the Dutch 2012 guidelines, even in more recent years, such as 2014, not even half of the eligible patients in fact underwent SLNB. When accounting for a possible delay to adoption of the 7th AJCC, SLNB enactment rose to 56.6% in 2014; however, there was an apparent 3-year delay from 2010 to 2013 due to patients with mitoses > 1/mm^2^ in whom SLNB was not performed. We found no studies on delays in the adoption of new guidelines in order to compare this finding.

We found female sex, older age, and melanoma in the head and neck region to be associated with non-enactment of SLNB. Huismans et al. assessed factors such as sex, age, socioeconomic status, BT, and hospital type influencing the use of SLNB in the north-eastern part of The Netherlands and found 42% of SLNB enactment in a total of 2413 patients with melanomas with a BT > 1 mm;^23^ however, compared with other nations, this percentage is low. Bilimoria et al.^24^ used US National Cancer Database (NCDB) data (*n* = 16,598) of stage I and II melanoma patients, between 2004 and 2005, and found a 48.7% enactment rate; Murtha et al.^25^ used Surveillance, Epidemiology, and End Results (SEER) data of 13,307 melanoma patients, between 2010 and 2012, with a 59.9% enactment rate; Moreno-Ramirez et al.^26^ analyzed 478 melanoma stage T1a–T4b patients in their center, with a 63.2% enactment rate; and Blakely et al.^27^ analyzed 865 melanoma patients, between 2005 and 2015, with a 93.2% enactment rate.

 In The Netherlands, considerable regional practice variation of 22.5–56.6% was previously reported by Verstijnen et al.^28^ in patients with a BT > 1 mm. Interesting is the finding that guidelines for SLNB enactment are not adequately adhered to. Explanations for this non-adherence in general can vary greatly, ranging from lack of familiarity to low outcome expectancy or disagreement.^29^ For melanoma-specific adherence to guidelines, Kang and Wong and Varey et al.^30^,^31^ showed that for wide local excisions for melanoma, surgeons with a high melanoma caseload (> 30) were more likely to perform procedures concordant with the guidelines than those with a lower caseload. Another reason might be that Dutch guidelines have only advised on SLNB since 2005, and waited until 2012 to provide a recommendation, which is still not solid advice. Other than that, we do not have a plausible explanation, other than more defensive versus selective attitudes that may differ per country, with The Netherlands apparently being more selective and with a relatively low adherence rate of 39.7%. In line with this, Cormier et al.^32^ used US SEER data and showed almost 10% of stage IA melanomas are overtreated when it comes to lymph node therapy, probably reflecting a more defensive attitude. Another important finding is that for both SLNB indicated and non-indicated melanomas, female patients had significantly lower odds of receiving an SLNB, with an OR of 0.78 and 0.80, respectively. While Huismans et al. and Verstijnen et al. corroborate our findings, with ORs of 0.86 and 0.85, respectively, it is surprising that none of the previously mentioned studies have considered patient sex in their analyses. There are three possible sex-related explanations that may account for our lower OR; (1) female melanoma patients have other characteristics that we did not include in our multivariable model; (2) sex-specific decision making, e.g. when female patients more often decline SLNB, or medical information is perceived differently; or (3) clinician-specific sex bias in approaching and informing female patients. No studies have been conducted in melanoma patients to support any of these explanations, however there is some general evidence of physician sex-related differences in both decision making and approach to patients.^33^–^36^

Another finding supported by previous literature is that head and neck melanomas had the lowest percentage of SLNB enactment.^23^–^25^,^29^ This may be explained by the technical challenge associated with localization, and also as lymphatic drainage can occur to multiple or bilateral sites, with the sentinel lymph node itself being relatively small.^37^ Furthermore, our finding that older patients more often refrain from SLNB is also sustained by others.^23^–^26^,^28^ An explanation for this could be relevant comorbidities influencing prognosis in older patients, or a more conservative approach in view of a generally lower life expectancy.

Although we assessed multiple factors associated with SLNB use, we did not take into consideration socioeconomic status, race, and regional practice variation, which have been shown to influence SLNB use.^23^–^25^,^28^ Another limitation is that mitosis status was missing in 41.6% of T1 melanomas. As a mitotic rate ≥ 1/mm^2^ implies SLNB indication in the 7th AJCC, this might have influenced the number of eligible patients for SLNB. As opposed to Verstijnen et al. and Huismans et al., we included ulceration since its presence means SLNB indication for T1 melanoma.^38^ Other strengths of our study include our large sample size and generalizability due to the nationwide cohort.

## Conclusion

There was an increasing trend in SLNB enactment for all melanoma stages, except T1a melanoma. Enactment of SLNB did not comply well with recommendations in (inter)national guidelines. Female sex, higher age, and melanoma in the head and neck region were associated with non-enactment of SLNB.

## References

[1] American Cancer Society. Skin Cancer. Available at: https://www.cancer.org/cancer/skin-cancer.html. Accessed 8 Jan 2018.

[2] The Netherlands Cancer Registry. Available at: https://www.cijfersoverkanker.nl/. Accessed 8 Jan 2018.

[3] Morton DL, Wen DR, Wong JH (1992). Technical details of intraoperative lymphatic mapping for early stage melanoma. Arch Surg..

[4] Kroon BB, Bergman W, Coebergh JW, Ruiter DJ. 2nd Revised consensus skin melanoma. De Nederlandse Melanoom Werkgroep. *Ned Tijdschr Geneeskd.* 1997;141(42):2015–9. 9550753

[5] Greene F, Page D, Fleming I (2002). AJCC cancer staging manual (6th edition).

[6] Balch CM, Buzaid AC, Soong SJ (2001). EANM-EORTC general recommendations for sentinel node diagnostics in melanoma. J Clin Oncol..

[7] Balch CM, Gershenwald JE, Soong SJ (2009). Final Version of 2009 AJCC Melanoma Staging and Classification. J Clin Oncol..

[8] Bichakjian CK, Halpern AC, Johnson TM (2011). Guidelines of care for the management of primary cutaneous melanoma. American Academy of Dermatology. J Am Acad Dermatol..

[9] Chakera AH, Hesse B, Burak Z (2009). EANM-EORTC general recommendations for sentinel node diagnostics in melanoma. Eur J Nucl Med Mol Imaging..

[10] Jansen L, Nieweg OE, Peterse JL, Hoefnagel CA, Valdes Olmos RA, Kroon BBR (2000). Reliability of sentinel lymph node biopsy for staging melanoma. Br J Surg..

[11] Madu MF, Wouters MW, van Akkooi AC (2017). Sentinel node biopsy in melanoma: Current controversies addressed. Eur J Surg Oncol..

[12] Seyed Jafari SM, Jackle P, Michel A, Angermeier S, Hunger R, Shafighi M (2016). Prognostic value of sentinel lymph node biopsy in melanomas of different Breslow’s thickness. Swiss Med Wkly..

[13] Morton DL, Thompson JF, Cochran AJ (2006). Sentinel-node biopsy or nodal observation in melanoma. N Engl J Med..

[14] Faries MB, Thompson JF, Cochran AJ (2017). Completion dissection or observation for sentinel-node metastasis in melanoma. N Engl J Med..

[15] van Everdingen JJ, van der Rhee HJ, Koning CC (2005). Guideline ‘Melanoma’ (3rd revision). Ned Tijdschr Geneeskd..

[16] The Dutch Melanoma Workgroup. The Dutch guideline melanoma 2016 (revised version). Available at: https://www.oncoline.nl/melanoma1. Accessed 8 Jan 2018.

[17] Veerbeek L, Kruit WH, de Wilt J, Mooi WJ, Bergman W (2013). Revision of the national guideline ‘Melanoma’ [in Dutch]. Ned Tijdschr Geneeskd..

[18] Gershenwald JE, Scolyer RA, Hess KR, et al. Melanoma staging: Evidence-based changes in the American Joint Committee on Cancer Eighth edition Cancer Staging Manual. *CA Cancer J Clin*. 2017;67(6):472–92. 10.3322/caac.21409 PMC5978683 29028110

[19] Oude Ophuis CM, van Akkooi AC, Rutkowski P (2016). Effects of time interval between primary melanoma excision and sentinel node biopsy on positivity rate and survival. Eur J Cancer..

[20] Gyorki DE, Barbour A, Hanikeri M, Mar V, Sandhu S, Thompson JF (2017). When is a sentinel node biopsy indicated for patients with primary melanoma? An update of the ‘Australian guidelines for the management of cutaneous melanoma’. Australas J Dermatol..

[21] National Institute for Health and Care Excellence (NICE) Guideline. Melanoma: Assessment and management. NICE; 2015. 26334080

[22] Coit DG, Thompson JA, Algazi A, et al. Melanoma, version 2.2016, NCCN clinical practice guidelines in oncology. *J Natl Compr Canc Netw*. 2016;14(4):450–73. 10.6004/jnccn.2016.0051 27059193

[23] Huismans AM, Niebling MG, Wevers KP, Schuurman MS, Hoekstra HJ (2014). Factors influencing the use of sentinel lymph node biopsy in The Netherlands. Ann Surg Oncol..

[24] Bilimoria KY, Balch CM, Wayne JD (2009). Health care system and socioeconomic factors associated with variance in use of sentinel lymph node biopsy for melanoma in the United States. J Clin Oncol..

[25] Murtha TD, Han G, Han D (2018). Predictors for use of sentinel node biopsy and the association with improved survival in melanoma patients who have nodal staging. Ann Surg Oncol..

[26] Moreno-Ramirez D, Tejera-Vaquerizo A, Mendonca FI, Ojeda-Vila T, Ferrandiz L (2017). Making decisions on sentinel lymph node biopsy for malignant melanoma: Prioritization of determinants using a decision tree. J Eur Acad Dermatol Venereol..

[27] Blakely AM, Comissiong DS, Vezeridis MP, Miner TJ (2018). Suboptimal compliance with National Comprehensive Cancer Network Melanoma guidelines: who is at risk?. Am J Clin Oncol..

[28] Verstijnen J, Damude S, Hoekstra HJ (2017). Practice variation in sentinel lymph node biopsy for melanoma patients in different geographical regions in The Netherlands. Surg Oncol..

[29] Cabana MD, Rand CS, Powe NR (1999). Why don’t physicians follow clinical practice guidelines?. A framework for improvement. JAMA..

[30] Kang R, Wong SL (2017). Melanoma surgery: Why don’t we let the guidelines guide practice?. Ann Surg Oncol..

[31] Varey AHR, Madronio CM, Cust AE (2017). Poor adherence to national clinical management guidelines: a population-based, cross-sectional study of the surgical management of melanoma in New South Wales. Australia. Ann Surg Oncol..

[32] Cormier JN, Xing Y, Ding M (2005). Population-based assessment of surgical treatment trends for patients with melanoma in the era of sentinel lymph node biopsy. J Clin Oncol..

[33] Ferguson MK, Huisingh-Scheetz M, Thompson K, Wroblewski K, Farnan J, Acevedo J (2017). The influence of physician and patient gender on risk assessment for lung cancer resection. Ann Thorac Surg..

[34] Hershman DL, Buono D, Jacobson JS (2009). Surgeon characteristics and use of breast conservation surgery in women with early stage breast cancer. Ann Surg..

[35] Borkhoff CM, Hawker GA, Kreder HJ, Glazier RH, Mahomed NN, Wright JG (2013). Influence of patients’ gender on informed decision making regarding total knee arthroplasty. Arthritis Care Res (Hoboken)..

[36] Hershman DL, Buono D, McBride RB (2008). Surgeon characteristics and receipt of adjuvant radiotherapy in women with breast cancer. J Natl Cancer Inst..

[37] Tanis PJ, Nieweg OE, van den Brekel MW, Balm AJ (2008). Dilemma of clinically node-negative head and neck melanoma: Outcome of “watch and wait” policy, elective lymph node dissection, and sentinel node biopsy: a systematic review. Head Neck..

[38] Balch CM, Soong SJ, Gershenwald JE (2001). Prognostic factors analysis of 17,600 melanoma patients: Validation of the American Joint Committee on Cancer Melanoma staging system. J Clin Oncol..

